# Making Symptoms Visible: The Impact of Real-Time PROM Integration in Pediatric Oncology

**DOI:** 10.3390/children13020164

**Published:** 2026-01-23

**Authors:** Natalie Bradford, Ethan Whalan, Paula Condon, Remziye Semerci, Alison Bowers, Xiomara Skrabal Ross

**Affiliations:** 1 School of Nursing, Queensland University of Technology, Brisbane, QLD 4101, Australia; ethan.whalan@hdr.qut.edu.au (E.W.); ap.bowers@qut.edu.au (A.B.); 2 Cancer and Palliative Care Outcome Centre at Centre for Children’s Health Research, Queensland University of Technology, Brisbane, QLD 4001, Australia; xiomara.skrabalross@qut.edu.au; 3 Cancer Council Queensland, Fortitude Valley, Brisbane, QLD 4006, Australia; 4 Nursing Research, Workforce Learning and Development, Centre for Children’s Health Research, Queensland Children’s Hospital and Health Service, Brisbane, QLD 4101, Australia; paula.condon@health.qld.gov.au; 5 School of Nursing, Koç University, Istanbul 34450, Türkiye; rsemerci@ku.edu.tr; 6 Canteen Australia, Sydney, NSW 2042, Australia

**Keywords:** Patient Reported Outcome Measures (PROM), pediatric oncology, child, neoplasms, symptom assessment, electronic health record, workflow integration, algorithms

## Abstract

**Highlights:**

**What are the main findings?**

- Children with cancer often experience severe symptoms that go undetected, including appetite changes, fatigue, nausea and vomiting and pain.
- Use of Patient-Reported Outcome Measures (PROMs) could address this gap, but such measures are rarely implemented in routine clinical practice.

**What are the implications of the main findings?**

- This study demonstrates the impact of routine PROM use on symptom detection and clinical workflow.
- Clinical algorithms enhance sensitivity for identifying symptoms but may increase alert burden highlighting the need for workflow and alert optimization.

**Abstract:**

**Background/Objectives:** Children undergoing cancer treatment experience multiple distressing symptoms that often go undetected in routine care. This study evaluated the potential impact of integrating the Symptom Screening in Pediatrics Tool (SSPedi) into clinical workflows, focusing on symptom detection and implications for service delivery. **Methods:** Seventy children (aged 4–18 years) receiving active treatment, and/or their caregivers completed SSPedi weekly for eight weeks (*n* = 479 completions). Medical records were audited for documentation of symptom assessments and symptom prevalence. SSPedi completions were categorized using a clinical algorithm (low, moderate, immediate concerns) and compared with score-only threshold. **Results:** The most bothersome symptoms were appetite changes (12%), fatigue (11%), nausea/vomiting (9%) and pain (9%). Severe bother detected by SSPedi was more frequent while hospitalized than at home (e.g., appetite changes 17% versus 9%). Documentation rates of severe symptoms in medical records were substantially lower than SSPedi reports—12% when SSPedi was completed at home and 49% when completed in hospital. Applying the clinical algorithm flagged 58% of SSPedi completions as an immediate concern in home and 63% in hospital, compared with score-only thresholds (31% at home and 17% in hospital). Algorithm-based alerts for immediate concerns would have triggered almost twice as many phone calls as score-based thresholds (168 vs. 91). **Conclusions:** Routine PROM integration could improve symptom detection and timely intervention. Clinical algorithms enhance sensitivity but increase alert burden, highlighting the need to review thresholds and redesign workflows.

## 1. Introduction

Patient Reported Outcome Measures (PROMs) provide an authentic insight into a patient’s health status, unfiltered by external interpretations, offering valuable information about the actual impact of disease and treatment from the patient’s perspective [1,2]. While PROMs have demonstrated improved outcomes for adults with cancer, their use to inform clinical care of children with cancer is not yet established [3].

Cancer treatment is inherently toxic, and all children undergoing therapy will experience a range of distressing symptoms including pain, nausea, fatigue, and psychological distress [4]. Chemotherapy often causes acute nausea and vomiting, radiotherapy frequently leads to persistent fatigue, and immunotherapy can produce a broad spectrum of immune-related symptoms with an unpredictable onset and intensity. Understanding the overall symptom burden from both the disease and different treatments is crucial to determining the most appropriate management strategy [5]. Both pharmacological (e.g., analgesics, antiemetics) and non-pharmacological interventions (e.g., mindfulness, physical activity, music therapy) can alleviate symptom distress [6]. However, their implementation depends upon timely recognition and reporting of symptoms. Evidence shows that children often struggle to articulate their experiences, and parents may misinterpret symptom severity, sometimes viewing these as an unavoidable part of cancer treatment, or even as evidence that the treatment is working [6].

PROMs, such as the Symptom Screening in Pediatrics (SSPedi), demonstrate effectiveness in improving symptom detection [6,7]. PROMs enable children and parents to accurately report the child’s experience, thereby facilitating appropriate interventions to address symptoms [8].

Implementing PROMs into routine care is a complex multi-level process influenced by organizational, technological and human factors [9]. Key considerations include integrating PROMs into workflows, ensuring documentation in medical records and establishing thresholds for concern and interventions. Thresholds are commonly based on PROM score alone; however, this may over- or underestimate the severity of individual symptoms [10]. In previous work, we developed clinical algorithms for SSPedi that provide a more nuanced approach by considering concern with individual symptoms, previous responses and diagnosis [11].

Our aim in this study was to evaluate the potential impact of implementing a PROM into routine care on (a) the detection of concerning symptoms and (b) the implications for clinical services of responding to the PROM using the clinical algorithm versus score-based thresholds.

## 2. Materials and Methods

### 2.1. Study Design and Participants

This study was part of a larger project reported elsewhere [11,12,13]. Seventy children receiving active cancer treatment (chemotherapy, radiotherapy, or immunotherapy) and their parent proxies were recruited from a tertiary children’s hospital oncology clinic. Eligibility criteria included the following:

- Child aged 4–18 years
- Receiving active treatment within one or two weeks of enrolment
- Caregiver provided informed consent.

### 2.2. Symptom Assessment Using SSPedi

SSPedi is a validated PROM for use in children with cancer 8–18 years old (or caregiver proxy 4–18 years) and assesses the degree of bother for 15 symptoms on a 5-point Likert scale (0, no bother to 4, extremely bothered). For this study, severe bother was defined as a score of 3 (a lot of bother) or 4 (extremely bothered) [7,8].

SSPedi data were collected weekly for eight weeks through an electronic survey, administered through REDCap (https://project-redcap.org/, accessed on 19 January 2026), and included information about where the SSPedi was completed (at home, at the hospital outpatient clinic, or inpatient). SSPedi could be completed by the child alone, caregiver and child together or caregiver alone. No specific feedback was provided to participants following completion, and clinical staff were not routinely informed about the SSPedi entries unless there was serious concern about patient safety, in which case clinical staff were notified by telephone.

SSPedi data were collated and downloaded from REDCap into Excel spreadsheets and entries were categorized based upon the location of SSPedi completion (home versus hospital/clinic). This distinction was important because children in hospital could receive symptom management interventions as part of routine care, which we anticipated would be reflected in medical record documentation. In contrast, children at home would have information documented about symptom bother only if parents contacted the hospital.

### 2.3. Medical Record Data Collection

Electronic medical records were systematically reviewed and audited to assess the documentation of symptom prevalence and associated management interventions. A children’s oncology research nurse (PC) reviewed medical records corresponding to each day SSPedi was completed, as well as the day prior and the day after following methods established by Tomlinson et al. [14]. Using descriptors provided in Supplementary Table S1, the nurse recorded any documented information about the assessment and presence of any of the 15 symptoms of interest.

### 2.4. Clinical Algorithm for Level of Concern

A clinical algorithm was developed to categorize SSPedi responses into three levels of concern:

- Low (no action required),
- Moderate (discuss with clinical team with next visit)
- Immediate (contact clinical team for advice)

Thresholds were informed by multidisciplinary consensus and considered the nature of individual symptoms, previous responses and diagnosis. Clinical staff highlighted that this was important, as there were concerns the threshold may otherwise be set too low or too high if the response to SSPedi was based upon score alone [11]. For example, a pain score of 2 (medium bother) triggered immediate concern warranting intervention, whereas feeling cranky or angry scored at 4 (extreme bother) only triggered immediate concern if reported twice within 14 days. This approach differs from score-only methods, the standard method to score SSPedi, which typically classifies 0 = low, 1–2 = moderate, and 3–4 = immediate concern. Using score-alone methods does not consider the specific nature of individual symptoms, or how they may change over time [8]. The algorithms were not used in real time with SSPedi completion but applied retrospectively to real-world SSPedi data to examine the potential impacts. The algorithms and the nursing actions for immediate concern alerts are provided in Supplementary Table S2.

### 2.5. Data Analysis

Descriptive statistics were used to summarize child and caregiver characteristics. Total symptom bother was calculated by summing each SSPedi entry (range 0–60) and a box and whisker plot was used to visualize the data over time. For each symptom, proportions were calculated comparing SSPedi reports with medical record documentation (numerator = documented symptom; denominator = SSPedi reports). Analyses were stratified by setting (home versus hospital) and by severity score. To estimate potential clinical impact, we compared the number of SSPedi completions that would have triggered immediate concern using the algorithm and score-only threshold. Analyses were descriptive only; no inferential testing was conducted.

## 3. Results

Seventy children and their caregivers were recruited through convenience sampling at the oncology outpatient clinic providing 479 SSPedi completions over eight weeks. Most children were aged 4–7 years (*n* = 29, 41.4%), followed by those aged 13–18 years (*n* = 22, 31.9%). Sex distribution among the children was relatively balanced with 52.8% female and 47.1% male. Caregivers were predominantly aged 40–49 years (*n* = 33, 47.1%) and were mostly female (84.2%).

In terms of time since diagnosis, 30% were diagnosed less than 3 months prior to recruitment, 47.1% were between 3 and 12 months, and the remaining 20% were diagnosed more than 12 months prior to the study. The children’s cancer types were mostly hematological (75.7%), while 24.2% were classified as solid tumors. Table 1 details the clinical and demographic characteristics of participating children and their caregivers.

### 3.1. Symptom Bother Reported by SSPedi

**Figure 1:**
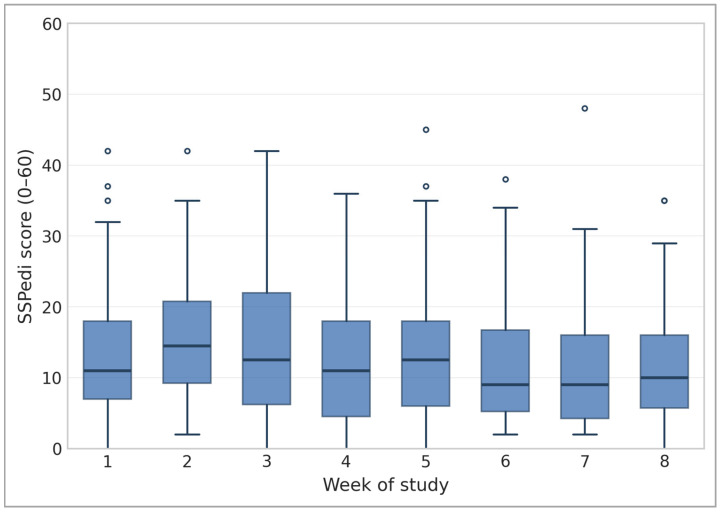
Box and Whisker Plot of Total symptom burden from 70 patients over 8 weeks.

Across the cohort, total symptom bother varied over time, with an initial rise over weeks two and three, followed by a gradual decline over the study period (Figure 1). The four most frequently reported bothersome symptoms (scores 3–4) were:

- Appetite changes (12.1%)
- Feeling tired (11.3%)
- Nausea/Vomiting (9%)
- Hurt or pain (8.8%)

There were no statistical differences in symptom bother associated with child age, sex, time since diagnosis, or cancer type.

### 3.2. Documentation of Symptom Assessment in Medical Records Compared with Prevalence Reported by SSPedi

Of the 479 SSPedi completions, 291 were completed at home and 188 in a hospital environment (including inpatient and outpatient clinic settings). Documentation in medical records was substantially lower than SSPedi self-reported symptom bother, even for severe symptoms (SSPedi scores 3–4) as shown in Table 2.

At home, only 12% of severe symptoms reported by SSPedi were documented in medical records. In hospital, documentation improved but remained incomplete, rarely exceeding 50% of reported bother. Pain and nausea/vomiting were most likely to be documented when severe, whereas psychological symptoms and peripheral neuropathy (tingly or numb hand or feet) were rarely recorded in medical records. In some cases, symptom prevalence was documented in medical records despite SSPedi indicating no symptom bother; this was more common in the hospital setting.

### 3.3. Algorithm Versus Score Based Concern Levels

Applying the clinical algorithm increased the proportion of SSPedi completions flagged as an immediate concern response (Table 3). In the home environment, the algorithm categorized 58% of SSPedi completions as an immediate concern and would have triggered 168 phone calls compared to score-only thresholds, which categorized 31% as immediate concern and would have triggered 91 calls. Based upon score alone, the majority (48%) of SSPedi completions at home were categorized as a moderate concern. This reflects the algorithm’s design to prioritize symptoms of clinical significance rather than severity score alone. In the hospital environment, an immediate concern would be triggered in 63% of completions using the algorithm, compared with 17% using score-only thresholds.

### 3.4. Estimated Impact on Clinical Services

**Figure 2:**
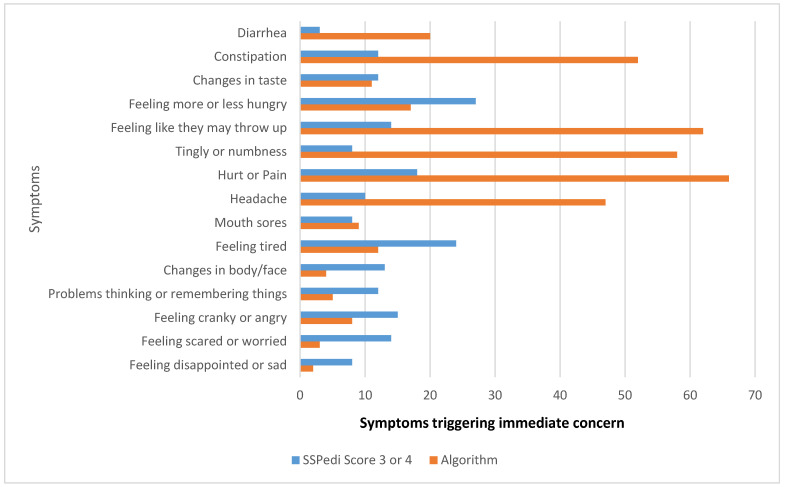
Count of symptoms triggering immediate concern determined by home completion of SSPedi by score versus algorithm (*N* = 291).

Figure 2 details the symptoms contributing to SSPedi immediate concern alerts in the home setting. The algorithm prioritized symptoms such as pain, tingly or numb hands or feet, and nausea, which were most likely to trigger phone calls, and deprioritized psychological symptoms unless they were sustained over multiple SSPedi reports.

## 4. Discussion

This study evaluated the potential impact of implementing a symptom PROM for children’s cancer into routine care focusing on symptom detection and implications for clinical workflow. Across 479 SSPedi completions, symptom bother varied over time, with appetite changes, fatigue, nausea/vomiting and pain emerging as the most frequent severe symptoms. These findings are consistent with the previous literature highlighting these symptoms as common and distressing for children during cancer treatment [3,15,16]. Findings also highlight that these symptoms often go undetected in routine clinical practice and thus unaddressed, leading to sustained bother and distress. All symptoms in SSPedi are amenable to intervention, and most have strong evidence supporting both pharmacological and non-pharmacological interventions [6,17]. However, interventions can only be offered to children if parents and clinicians are aware of the magnitude of bother children are experiencing and the most appropriate intervention.

### 4.1. Symptom Detection and Documentation

Self-report symptoms via SSPedi identified substantially higher prevalence and bother compared with documentation in medical records for both children in a home environment and those in hospital. For example, pain rated ‘a lot’ (score 3) or ‘extremely’ (score 4) was documented in medical records for only 22% of home completions and 62.5% of hospital completions. Bother from tingly or numb hands and feet, representing peripheral neuropathy was not documented in medical records at all. These discrepancies mirror findings from other studies that report poor correlation—often less than 60%—between self-reported symptoms and medical record documentation [14,18,19]. While we found that symptoms reported as extremely bothersome were more likely to be documented in medical records, particularly when a child was hospitalized, a substantial number of children may still experience distress from symptoms that are unnoticed and untreated. This reinforces the need for systematic PROM integration across care settings to ensure timely recognition and intervention [20].

Several factors may explain the low documentation rates. First, as an exploratory study, SSPedi scores were not shared with the clinical team in real time, limiting awareness of symptom burden [11]. Second, documentation in medical records may not accurately reflect actual clinical practice. An assessment of symptom burden and the provision of an intervention can occur without being documented [21]. Healthcare professionals must prioritize tasks, and documentation is just one of many tasks involved in providing care. Fatigue and burnout are increasingly common in children’s cancer care, which may further reduce documentation [22]. Additionally, the complexity of electronic medical records and the absence of automated prompts can lead to delays or omissions in entering records. These challenges are not unique to this setting but are prevalent across healthcare systems, particularly in settings where multiple care providers are involved [21].

### 4.2. Algorithm Versus Score-Based Thresholds

Applying the clinical algorithm substantially increased the proportion of SSPedi completions flagged for immediate concern compared to score-only thresholds (58% vs. 9% at home; 63% vs. 17% in hospital). The algorithm was developed in close consultation with a wide multidisciplinary team, including medical, pharmacy, nursing, social work, psychology, physiotherapy and dietetics [12]. This team stressed the importance of early identification of symptoms, so that appropriate interventions could be initiated early for clinically significant symptoms. For example, the physiotherapy team highlighted that referrals to manage peripheral neuropathy often came late; hence, the threshold for an immediate concern alert was set at ‘medium bother’ (score 3). A pain score of 2 triggered immediate concern, whereas psychological symptoms required sustained bother before escalation. Based on this approach, 167 ‘immediate concern’ alerts would have been generated by prompting parents to telephone the clinical team. This was twice the number of alerts that would have been generated by score-alone thresholds. This highlights the difference between the two methods in terms of sensitivity, specificity and response criteria. The algorithm, with its broader set of considerations, is more proactive in generating alerts. However, the higher sensitivity of the algorithm could also lead to an increase in false positives or unnecessary interventions, which may burden healthcare providers and resources [23]. In contrast, relying on score alone would trigger fewer alerts of immediate concern but runs the risk of missing early signs of distress in patients who could benefit from earlier intervention.

This raises an important question regarding the trade-off between sensitivity, specificity and the practical implications for patient care. Further research is needed to evaluate the effectiveness of both approaches in a real-world setting, considering not only the number of alerts, but also the clinical outcomes associated with each method. Furthermore, it is essential to understand the patient and clinician perspectives on the frequency of alerts and interventions, which could provide valuable insight into achieving an optimal balance between proactive monitoring and avoidance of alert fatigue [23]. Future studies may also consider exploring how to combine elements from both the algorithm and the SSPedi score system to offer a more nuanced and efficient approach to clinical alerts and care.

### 4.3. Limitations

This exploratory study did not provide real-time SSPedi data to clinicians, which likely reduced awareness of individual symptoms and, in turn, documentation rates. We applied the clinical algorithm retrospectively to SSPedi data, which limits generalizability and conclusions about its real-time feasibility. Retrospective analysis allowed us to estimate alert frequency and workflow implications but did not test integration into clinical systems or clinical response. The study was undertaken in a single tertiary center, and the use of a convenience sample further limited generalizability. Documentation practices vary between clinicians, and medical records may not fully capture symptom assessments, experience, or interventions provided. The estimates provided here should, therefore, be interpreted with caution.

### 4.4. Clinical and Research Implications

Symptom assessment and management are cornerstones of high-quality cancer care, and routine use of PROMs like SSPedi can improve detection and prompt timely interventions, reducing distress from unmanaged symptoms [24,25]. However, real-time deployment would require embedding SSPedi within the electronic health record, with an automated algorithm-based triage system and defined escalation pathways. Training programs that incorporate comprehensive symptom assessment, management, intervention and documentation are essential to optimize care quality and close gaps in symptom management [6]. These goals can be achieved through clinical workshops and proactive education. Integration of clinical decision support systems could further enhance responsiveness while mitigating alert fatigue [20]. Workflow changes could include assigning responsibility for monitoring alerts to a nurse coordinator, integrating symptom review into ward rounds and consultations, and documenting interventions in real time.

Future research should evaluate the impact of real-time PROM integration on clinical outcomes, caregiver satisfaction and healthcare resource utilization across different settings including home, ambulatory care, and hospital. Studies should also examine clinician perspectives on technical integration, including alert frequency and response time, as well as develop strategies to optimize thresholds for interventions that do not overwhelm clinical teams. The clinical algorithm should be refined in light of these findings to ensure it remains relevant and appropriate to clinical practice. Understanding these phenomena is essential for optimizing symptom control for children with cancer.

## 5. Conclusions

Routine integration of patient-reported outcomes measures such as the SSPedi into pediatric oncology care has the potential to significantly improve symptom detection and timely intervention. This study demonstrates that self-reported symptom burden is substantially higher than that documented in medical records, particularly in home-care settings, highlighting a critical gap in current clinical practice. While clinical algorithms can enhance sensitivity for identifying symptoms of concern, they also raise challenges related to alert fatigue and resource allocation. Future research should focus on real-time PROM implementation, optimizing alert thresholds and evaluating the impact on clinical outcomes and caregiver satisfaction. Embedding PROMs into electronic health records and supporting clinicians through training and workflow redesign will be essential to ensure that symptom management is practical, comprehensive and person-centered.

## Figures and Tables

**Table 1:** Clinical and demographic characteristics of participants (*N* = 70).

Variable	Participants (***n***)	% (All Participants)
Child Age Group (years)		
2–3	11	15.7
4–7	29	41.4
8–12	8	11.4
13–18	22	31.4
Child Sex		
Male	33	47.1
Female	37	52.8
Caregivers’ Age Group (years)		
18–39	30	42.8
40–49	33	47.1
50–69	7	10.0
Caregivers’ Sex		
Male	10	14.2
Female	59	84.2
Unknown	1	1.6
Time Since Diagnosis		
<3 Months	21	30.0
3–12 Months	33	47.1
>12 Months	14	20.0
Unknown	2	2.9
Child Cancer Type		
Blood	53	75.7
Solid	17	24.2

**Table 2:** Documentation rate of SSPedi severe self-report symptoms.

SSPedi Domain and Score		Home *n* = 291 (%)			Hospital *n* = 188 (%)		
		A Documented	B SPPedi Score 3 or 4	A/B Rate %	C Documented	D SPPedi Score 3 or 4	C/D Rate %
Feeling sad or disappointed							
		2	8	25%	9	14	64%
Feeling scared or worried							
		3	14	21%	10	20	50%
Feeling cranky or angry							
		2	15	13%	4	20	20%
Problems with thinking or remembering things							
		0	12	-	1	5	20%
Changes in how your body or face looks							
		0	6	-	1	8	13%
Feeling tired							
		2	24	8%	14	30	47%
Mouth sores							
		2	8	25%	1	3	33%
Headache							
		1	10	10%	4	11	36%
Hurt or Pain							
		4	18	22%	15	24	63%
Tingly or numb hands or feet							
		0	8	-	0	4	-
Throwing up or feeling like you might throw up (vomiting or nausea)							
		3	14	21%	24	29	83%
Feeling more or less hungry than usual (changes in appetite)							
		3	27	11%	17	31	55%
Changes in taste							
		0	22	-	2	19	11%
Constipation (hard to poop)							
		1	12	8%	5	10	50%
Diarrhea (watery runny poop)							
		1	3	33%	6	10	60%

Note rate = Documented ÷ SSPedi self-report × 100.

**Table 3:** Categorization levels of concerns with individual symptoms determined by the clinical algorithm or score for the hospital and home environment.

	Clinical Algorithm	%	Score	%
**Home environment** *n* = 291				
Low concern	63	22%	61	21%
Moderate concern	60	21%	139	48%
Immediate concern	168	58%	91	31%
**Hospital environment** *n* = 188				
Low concern	44	23%	54	29%
Moderate concern	25	13%	103	55%
Immediate concern	119	63%	31	17%

## Data Availability

The data presented in this study are available on request from the corresponding author, with the appropriate approvals aligning with the Ethics approval for this study.

## Supplementary Materials

The following supporting information can be downloaded at: https://www.mdpi.com/article/10.3390/children13020164/s1, Table S1: Descriptors of symptoms from medical records; Table S2 Clinical Algorithms and nursing actions for immediate concern.

## Abbreviations

The following abbreviations are used in this manuscript:

PROM	Patient Reported Outcome Measure
SSPedi	Symptom Screening in Pediatrics
