# Supplementary material for: Making Symptoms Visible: The Impact of Real-Time PROM Integration in Pediatric Oncology
Source: Children (Basel). 2026 Jan 23;13(2):164. doi: 10.3390/children13020164 (PMC12939280; doi:10.3390/children13020164)
Supplement: Supplementary file 1 [file children-13-00164-s001.zip › children-4060413-supplementary.pdf]

Descriptor of Symptoms in Medical Records

Symptom	Example of Documentation
Sad/Disappointed	Unhappy, mood flat, upset, very upset, cry/cried/crying (and squirmed) crying inconsolably, unsettled, miserable, sooky, bit bleh, mood down
Scared/Worried	Scared, worried, lots of worry, dread, anxiety, anxious, incredibly anxious, distressed, nervous, wary, apprehensive, gasping for breath and increased heart rate, physical avoidance, refusing procedure, reluctant, phobic
Cranky/angry	Agitated, grumbly, annoyed, grumpy, grouchy, cranky, yelling, irritable, over it, over being in hospital
Thinking/Remembering	Confusion, delirium
Changes in how body/face Look	Look, people look at me funny, skin dry, erythematous, dry skin, transient redness, hirsutism
Fatigue	Tired, lethargic, lethargy, bleh, very tired, exhaustion, fatigued, very sleepy, sleeping most of the time, drowsy throughout day, wanting to sleep, spending lots of time sleeping, looking more tired, general malaise, struggling energy levels, weak
Mouth Sores	Gums inflamed, sore mouth, sore throat
Headache	Headache (above eyes)
Hurt/Pain	Sore, pain, discomfort, tender, tenderness, generalised tenderness, breakthrough, feels sharp pain (intermittent), ache/s, hurts, hurting, feels like crap, cramps, sensitive/sensitivity, pain score, miserable with pain, worse than usual,
Tingly/Numb	Funny feeling in hands/feet. Dropping things
Throwing up/Nausea	Nausea, wants to eat but feels unwell, vomit, vomited, vomiting, gagged, gagging, feeling unwell, vomited ++, persistent nausea, nauseous, sick in tummy, unable to tolerate feeds
Hungry/Appetite	Poor oral intake, minimal oral intake, not eaten, sips only, eating small amounts, reduced oral intake, eating <25% of usual intake, refusing oral intake, reluctant to drink, Small appetite, no appetite, some oral intake, reduced/slow rate/s nasogastric feeds, off food, not interested in oral diet, not interested in eating, refusing food/fluids
Taste	Taste changes
Constipation	Dry, hard, BNO regularly, No BM, difficult BO, constipation, straining,
Diarrhoea	Diarrhoea, BO accidents, open multiple times, loose stools, runny, watery

Symptom algorithm for thresholds and immediate concern nursing actions

<table>
<tr><th>Symptoms</th><th>Threshold
(Single entry)</th><th>Threshold
(14-day entry)</th><th>Immediate concern and appropriate nursing action</th></tr>
<tr><td>1. Feeling disappointed or sad</td><td rowspan="7">T1 ≤ 3 = low concern

T1 = 4 = moderate concern</td><td rowspan="7">T1 ≤ 3 and T2 ≤ 1 = low concern

T1 = 2 and T2 = 2 = moderate concern

T1 ≥ 3 and T2 ≥ 3= immediate concern</td><td rowspan="2">Concern eith bother with psychological symptoms. Assess for psychological distress and consider causes- e.g., steroids. Consider the need for further assessment and allied health support (e.g. psychology or occupational therapy).
Consider non-pharmacological interventions.
Document interactions in iEMR</td></tr>
<tr><td>2. Feeling scared or worried</td></tr>
<tr><td>3. Feeling cranky or angry</td><td>Concern with bother with cognition.
Assess for potential causes, including neurological causes. Consider the need for referral for further assessment and allied health support.
Document interactions in iEMR</td></tr>
<tr><td>4. Problems with thinking or remembering things</td><td rowspan="2">Concern with bother with body image.
Assess for psychological distress. Consider the need for referral for further assessment and allied health support. Consider non-pharmacological interventions.
Document interactions in iEMR</td></tr>
<tr><td>5. Changes in how your body or face look</td></tr>
<tr><td>6. Feeling more or less hungry than you usually do</td><td>Concern with bother with appetite.
Assess potential problems associated with nausea/vomiting, constipation, and medications (steroids). Consider the need for further assessment and referral to dieticians and other allied health support.
Document interactions in iEMR.</td></tr>
<tr><td>7. Changes in taste</td><td>Concern with bother with changes in taste. Acknowledge this is an unpleasant symptom and reassure that this is transient and will pass.
Document interactions in iEMR.</td></tr>
<tr><td>8. Fatigue</td><td>T1 ≤ 2 = low concern

T1 = 3 = moderate concern

T1 = 4 = immediate concern</td><td>T1≤ 2 and T2 ≤ 1 = low concern

T1 ≤2 and T2 ≤ 2 = moderate concern

T1 ≥ 3 and T2 ≥ 3= immediate concern</td><td>Concern with the level of bother from fatigue. While fatigue is a common symptom- consider potential causes, including anemia, bleeding, fluid imbalance, and psychological causes. Consider the need for further assessment, including medical if indicated. Consider non-pharmacological interventions.
Document interactions in iEMR</td></tr>
<tr><td>9. Mouth sores</td><td>T1 ≤ 1 = low concern

T1 = 2 = moderate concern

T1≤ 3 = immediate concern</td><td>T1 ≤ 1 and T2≤1 = low concern

T1=2 and T2≤2 = immediate concern</td><td>Concern with mucositis.
Assess potential causes, e.g. chemotherapy, radiotherapy and need for further assessment, including oral intake, and medical review for pain relief if indicated.
Document interactions in iEMR</td></tr>
</table>

Symptom algorithm for thresholds and immediate concern nursing actions

10. Headache	T1 ≤ 1 = **low concern**<br><br>T1 ≥ 2 = **immediate concern**<br><br>IF Brain/spinal tumours<br><br>T1 = 0 = **low concern**<br><br>T1 ≤ 1= **immediate concern**	T1 ≤ 1 and T2 ≤ 1 = **low concern**<br><br>T≤ 2 and T2 ≥ 2 = **immediate concern**<br><br>IF brain/spinal tumour<br><br>T1 = 0 and T2 = 0 = **low concern**<br><br>T1 ≤ 1 and T2 ≥ 1 = **immediate concern**	**Concern with headache.**<br>Assess potential causes and use of prescribed analgesics to manage pain. Consider the need for further assessment/ review by medical staff.<br><br>**IN CHILDREN WITH BRAIN TUMOURS**<br>Consider neurological causes for headaches, including shunt malfunction. Assess and consider the need for further assessment or referral, including medical review.<br>*Document interactions in iEMR*
11. Hurt or pain (other than a headache)	T1 ≤ 1 = **low concern**<br><br>T1 ≤ 3 = **moderate concern**<br><br>T1 =4 = **immediate concern**	T1 ≤ 0 and T2 = 0 = **low concern**<br><br>T1 ≤ 3 and T2 ≤ 1 = **moderate concern**<br><br>T1 ≤ 4 and T2 ≥ 2 = **immediate concern**	**Concern with pain.**<br>Assess potential causes and use of prescribed analgesics to manage pain. Consider non-pharmacological interventions. Consider the need for further assessment or medical review.<br>*Document interactions in iEMR*
12. Tingly or numb hands or feet	T1 = 0 = **low concern**<br><br>T1 ≥ 3 = **moderate concern**<br><br>T1 =4 = **immediate concern**	T1 = 0 and T2 = 0 = **low concern**<br><br>T1 ≥ 1 and T2 ≥ 1 = **immediate concern**	**Concern with altered sensation.**<br>Assess potential causes – e.g., chemotherapy-induced peripheral neuropathy or neurological complications. Consider the need for referral to physiotherapy and medical review.<br>*Document interactions in iEMR*
13. Throwing up or feeling like you may throw up			**Concern with nausea/vomiting.**<br>Assess potential causes including chemotherapy induced. Assess the use and effectiveness of prescribed anti-emetics and fluid balance. Consider non-pharmacological interventions. Consider the need for medical review.<br>*Document interactions in iEMR*
14. Constipation	T1 ≤ 1 = **low concern**<br><br>T1 ≥ 2 = **immediate concern**	T1≤ 1 and T2≤ 1= **low concern**<br><br>T1 ≤ 2 and T2 ≤ 2 = **moderate concern**	**Concern with constipation.**<br>Assess potential causes, including chemotherapy-induced. Assess the use of aperients as prescribed. Consider need for a dietician or medical review.<br>*Document interactions in iEMR*
15. Diarrhoea			**Concern with diarrhea.**<br>Assess potential causes and consider fluid balance and skin integrity in a child. Consider the need for a dietitian or medical review.<br>*Document interactions in iEMR*
